# Asymptomatic Delayed Coil Migration from an Intracranial Aneurysm: A Case Report

**DOI:** 10.1155/2011/901925

**Published:** 2011-09-29

**Authors:** Anirban Deep Banerjee, Leopoldo Guimaraens, Hugo Cuellar

**Affiliations:** ^1^Department of Neurosurgery, Louisiana State University Health Sciences Center, P.O. Box 33932, Shreveport, LA 71130-33932, USA; ^2^Department of Endovascular Therapy, General Hospital of Catalonia, 08174 Barcelona, Spain

## Abstract

*Objective*. To describe asymptomatic delayed migration of a coil loop in a patient following successful coil embolization of an anterior communicating artery saccular aneurysm. *Methods*. A 24-year-old man with a ruptured anterior communicating artery saccular aneurysm underwent coil embolization with one helical ultrasoft coil. *Results*. A followup CT scan head and a cerebral angiogram one month following the procedure revealed distal migration of an intra-aneurysmal coil loop into the left pericallosal artery. The patient, however, remained asymptomatic. *Conclusion*. Delayed migration of coil following embolization of an intracranial aneurysm is an extremely rare occurrence. An asymptomatic presentation, as in our patient, is even more unique. The stent-like configuration of the migrated spiral coil loop probably prevented complete occlusion of the blood vessel.

## 1. Introduction

Delayed coil-loop migration is a rare complication of coil embolization of intracranial aneurysms, with few reported cases till date [1–5], usually presenting with significant morbidities. We report an unusual occurrence of asymptomatic presentation of such an event. 

## 2. Case Illustration

**Figure 1:**
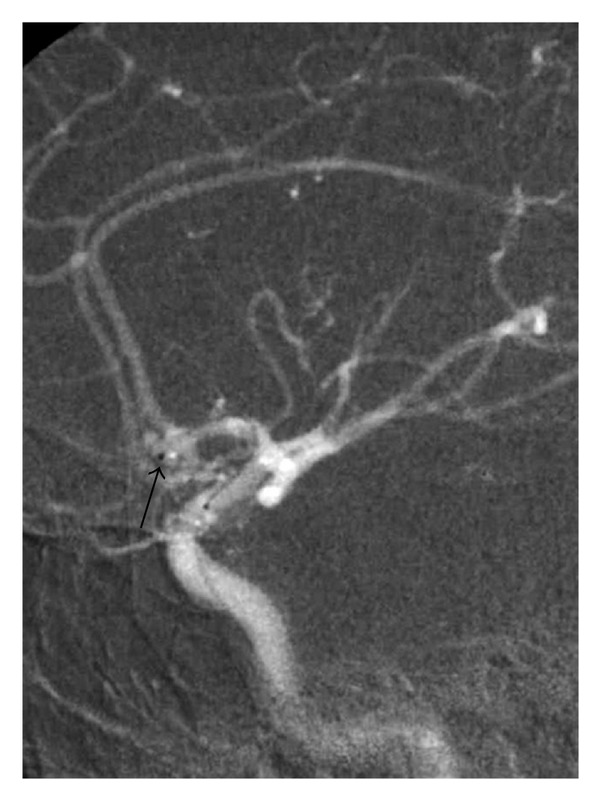
Left ICA road map during aneurysm coiling showing the tip of the microcatheter inside the Acom aneurysm. Note the elongated shape of the aneurysm (arrow).

A 24-year-old man presented sudden onset severe headache. Neurological examination revealed no deficits. Noncontrast head CT revealed subarachnoid hemorrhage. A cerebral angiogram demonstrated a saccular aneurysm in the region of the anterior communicating artery measuring approximately 5 mm in height and 2 mm in width (fundus) with a neck measuring 2 mm (Figure 1). Patient underwent uneventful coil embolization with one helical GDC ultrasoft coil 2 mm × 8 cm (Boston Scientific). Complete aneurysmal occlusion was achieved after one coil, with no evidence of intraprocedural coil migration. After procedure the patient had an uneventful hospital stay and was discharged one week after. At discharge, the Glasgow Outcome Score was 5.

**Figure 2:**
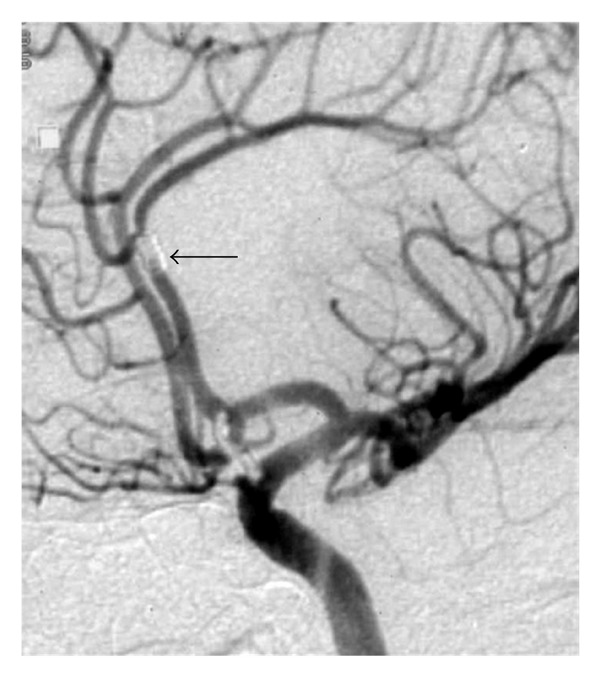
Left ICA DSA after aneurysm has been clipped showing that the helical coil (arrow) is in the pericallosal artery with good distal blood flow.

Routine followup at one month revealed no deficits. However, a followup head CT raised suspicion of coil migration. There was no evidence of cerebral ischemia. Subsequently, cerebral angiography confirmed interval migration of an intra-aneurysmal coil loop into the left pericallosal artery. However, there was good distal blood flow. Patient decided to undergo clipping of the aneurysm instead of a second coil embolization. Surgical clipping of the aneurysm was uneventful (Figure 2).

## 3. Discussion

Delayed interval migration of coil loops is rare complication of coil embolization procedures for intracranial aneurysms. These events have been usually noted in the event of coil mismatch due to unfavorable neck-to-fundus ratio. In the case described by Fiorella et al., the aneurysm was small (4.5 × 4.4 × 3.9 mm) with an unfavorable neck-fundus ratio (neck measured 2.5 mm) [1]. Other cases, like the ones described by both Gao et al. and Motegi et al. also featured broad-wide necked aneurysms treated using ballon or stent assisted techniques [2, 4]. However, our patient harbored an aneurysm of favorable neck-fundus ratio and no adjunct to assisted technique was necessary. In our case, the aneurysm was long and narrow, and a helical coil in the same shape was thought to be a good option. After embolization, the coil appeared stable and completely embolize; we think that maybe the aneurysm may have been larger than apparent, and the ultrasoft coil eventually was dragged out of the aneurysm by the blood flow.

Time periods from the initial procedure to the detection of coil migration range from a few days to several months [1, 2, 4], although the time of actual migration remains speculative at best. Gao et al. suggest that coil migration occurred while their patient was receiving antiplatelet therapy because a layer of neointima was already covering the migrated coil at followup, 5 months after coiling.

Coil migration usually leads to ischemic complications. However, Gao et al. have reported a case in which followup angiography 5 months later revealed that a coil had escaped the confinement of stent-assisted coiled aneurysm and migrated distally into the middle cerebral artery territory without occluding any arterial branches or causing symptoms. As in our patient, they had used 2D ultrasoft (helical) coils as well [2]. We hypothesize that unlike a solid 3D coil, the stent-like configuration of the migrated spiral coil loop provided a conduit for continued blood flow in both these patients. This factor might have prevented complete occlusion of the blood vessel, thus accounting for the asymptomatic nature of the event.

## 4. Conclusion

Delayed migration of coil loop following embolization of an intracranial aneurysm is an extremely rare occurrence but not invariably morbid, as evident in our patient. It can occur even in aneurysms with favorable neck-fundus ratio. The stent-like configuration of the migrated spiral coil loop probably prevented complete occlusion of the artery and consequent ischemic complications.
